# Supplementary material for: A Critical View of the Application of the APEX Software (Aqueous Photochemistry of Environmentally-Occurring Xenobiotics) to Predict Photoreaction Kinetics in Surface Freshwaters
Source: Molecules. 2019 Dec 18;25(1):9. doi: 10.3390/molecules25010009 (PMC7017383; doi:10.3390/molecules25010009)
Supplement: Supplementary file 1 [file molecules-25-00009-s001.zip › 3.2.4_gcc-4.4.0.zip!/3.2.4_gcc-4.4.0/3.2.4_gcc-4.4.0/doc/octave/html/interpreter/Working-with-Matrices-and-Arrays-in-Mex_002dFiles.html]

Working with Matrices and Arrays in Mex-Files - Untitled


Next: Character Strings in Mex-Files,
Previous: Getting Started with Mex-Files,
Up: Mex-Files


---

#### A.2.2 Working with Matrices and Arrays in Mex-Files

The basic mex type of all variables is `mxArray`. All variables,
such as matrices, cell arrays or structures are all stored in this basic
type, and this type serves basically the same purpose as the
octave\_value class in oct-files. That is it acts as a container for the
more specialized types.

The `mxArray` structure contains at a minimum, the variable it
represents name, its dimensions, its type and whether the variable is
real or complex. It can however contain a number of additional fields
depending on the type of the `mxArray`. There are a number of
functions to create `mxArray` structures, including
`mxCreateCellArray`, `mxCreateSparse` and the generic
`mxCreateNumericArray`.

The basic functions to access the data contained in an array is
`mxGetPr`. As the mex interface assumes that the real and imaginary
parts of a complex array are stored separately, there is an equivalent
function `mxGetPi` that get the imaginary part. Both of these
functions are for use only with double precision matrices. There also
exists the generic function `mxGetData` and `mxGetImagData`
that perform the same operation on all matrix types. For example

```
     mxArray *m;
     mwSize *dims;
     UINT32_T *pr;
     
     dims = (mwSize *) mxMalloc (2 * sizeof(mwSize));
     dims[0] = 2;
     dims[1] = 2;
     m = mxCreateNumericArray (2, dims, mxUINT32_CLASS, mxREAL);
     pr =  = (UINT32_T *) mxGetData (m);
```

There are also the functions `mxSetPr`, etc., that perform the
inverse, and set the data of an Array to use the block of memory pointed
to by the argument of `mxSetPr`.

Note the type `mwSize` used above, and `mwIndex` are defined
as the native precision of the indexing in Octave on the platform on
which the mex-file is built. This allows both 32- and 64-bit platforms
to support mex-files. `mwSize` is used to define array dimension
and maximum number or elements, while `mwIndex` is used to define
indexing into arrays.

An example that demonstration how to work with arbitrary real or complex
double precision arrays is given by the file mypow2.c as given
below.

```
```
     #include "mex.h"
     
     void
     mexFunction (int nlhs, mxArray* plhs[], int nrhs, 
     	     const mxArray* prhs[])
     {
       mwIndex i;
       mwSize n;
       double *vri, *vro;
       
       if (nrhs != 1 || ! mxIsNumeric (prhs[0]))
         mexErrMsgTxt ("expects matrix");
     
       n = mxGetNumberOfElements (prhs[0]);
       plhs[0] = (mxArray *) mxCreateNumericArray 
         (mxGetNumberOfDimensions (prhs[0]),
          mxGetDimensions (prhs[0]), mxGetClassID (prhs[0]),
          mxIsComplex (prhs[0]));
       vri = mxGetPr (prhs[0]);
       vro = mxGetPr (plhs[0]);
     
       if (mxIsComplex (prhs[0]))
         {
           double *vii, *vio;
           vii = mxGetPi (prhs[0]);
           vio = mxGetPi (plhs[0]);
     
           for (i = 0; i < n; i++)
     	{
     	  vro [i] = vri [i] * vri [i] - vii [i] * vii [i];
     	  vio [i] = 2 * vri [i] * vii [i];
     	}
         }
       else
         {
           for (i = 0; i < n; i++)
     	vro [i] = vri [i] * vri [i];
         }
     }
```
```

with an example of its use

```
     b = randn(4,1) + 1i * randn(4,1);
     all(b.^2 == mypow2(b))
     => 1
```

The example above uses the functions `mxGetDimensions`,
`mxGetNumberOfElements`, and `mxGetNumberOfDimensions` to work
with the dimensions of multi-dimensional arrays. The functions
`mxGetM`, and `mxGetN` are also available to find the number
of rows and columns in a matrix.
